# Genetic Diversity of CRESS DNA Viruses in Rodent Virome

**DOI:** 10.3390/v18101128

**Published:** 2026-10-11

**Authors:** Igor Babkin, Viktor Panov, Sergey Tkachev, Valeria Fedorets, Nina Tikunova

**Affiliations:** 1 Laboratory for Molecular Microbiology, Institute of Chemical Biology and Fundamental Medicine SB RAS, 630090 Novosibirsk, Russia; sergey.e.tkachev@gmail.com (S.T.); f.valeriya41@gmail.com (V.F.); 2 Laboratory of Ecology of Communities of Vertebrate Animals, Institute of Systematics and Ecology of Animals SB RAS, 630091 Novosibirsk, Russia; panovv53@mail.ru

**Keywords:** CRESS DNA viruses, alphasatellites, virome, taxonomy, high-throughput sequencing

## Abstract

CRESS DNA viruses belong to a widely distributed and highly diverse group of viruses, which are often found during the analysis of viromes. However, data on their genetic diversity are still incomplete. The intestinal viromes of widespread vole species—northern red-backed vole and tundra vole—were sequenced. Based on the Rep protein sequence, 22 sequences of various CRESS DNA viruses and alphasatellites were identified; 14 of them were complete genomes. According to the genome organization ten isolates were assigned to CRESS DNA viruses of known families of the *Cressdnaviricota* phylum, and one isolate to the *Alphasatellitidae* family. Three complete genomes were unique and possibly belong to new viral families. Most sequences were assigned to the *Vilyaviridae* family of the *Cirlivirales* order. An unusual genome from the clade of regular CRESS DNA viruses but without the *cp* gene was identified, which suggests that it is a possible alphasatellite. This fact raises the question of revising the current paradigm of dividing Circular Rep-Encoding DNA sequences into viruses and alphasatellites. Two pairs of similar CRESS DNA genomes were detected in different rodent species, which indicates the need for further study of diversity and host specificity of CRESS DNA viruses.

## 1. Introduction

The use of a metagenomic approach to the study of viral communities has revealed a huge diversity of mammalian viruses [1,2,3,4]; in particular, the application of this technology to study rodent viromes has made it possible to detect a wide range of new viruses [5,6,7,8]. A significant part of the viruses detected in metagenomic studies belong to Circular Rep-Encoding Single-Strand DNA (CRESS DNA) viruses (*Cressdnaviricota* phylum, *Shotokuvirae* kingdom) [9,10,11,12]. To date, the *Cressdnaviricota* phylum is divided into two classes: *Arfiviricetes* and *Repensiviricetes*. The *Repensiviricetes* class (33,582 sequences in GenBank) contains a single *Geplafuvirales* order comprising three families. The *Arfiviricetes* class (35,382 sequences in GenBank, accessed on 1 March 2026) includes 12 orders and 21 families (ICTV; https://ictv.global/taxonomy, accessed on 1 March 2026).

CRESS DNA viruses have small, circular ssDNA genomes 1–6 kilobases long, and most of these viruses contain only two genes encoding the replication initiation protein called replicase (Rep) and a capsid protein (CP) [13]. Their genomes differ depending on the position of the replication *ori*, as well as the location and orientation of these genes within the genomes [14]. These viruses are often associated with alphasatellites—satellite DNA molecules that require the assistance of a helper virus for their encapsidation. The alphasatellite genomes encode a Rep protein similar to that of CRESS DNA viruses, but not the CP [15,16,17].

Replication of CRESS DNA viruses and alphasatellites occurs via a rolling circle mechanism and is initiated by a replicase (Rep protein) that binds to the specific *ori* sequence containing a characteristic nonanucleotide motif at the apex of the hairpin structure where replication begins [14,18]. Rep is a superfamily 3 helicase consisting of two main domains: the HUH endonuclease domain (His-hydrophobic-His motif) and the SF3 domain [13,19]. The HUH endonuclease domain of the Rep protein includes three conserved motifs known as rolling circle replication (RCR) motifs I, II, and III. The RCR I motif is involved in double-stranded DNA binding, the RCR II motif coordinates divalent metal ions, and the RCR III motif has endonuclease activity [12,14]. Notably, the *Genomoviridae* family genomes have an additional conserved Geminivirus Rep-like Sequence (GRS) motif [12,20]. The C-terminal SF3 helicase domain of all Rep proteins contains three conserved motifs: Walker-A, Walker-B, and motif C [12,21,22]. Rep protein sequences are used for taxonomic assignment of the CRESS DNA viruses.

CRESS DNA viruses are widespread on Earth from deep-sea samples to human lungs, where they may play an important role in different ecosystems [13,23,24,25,26]. Given their possible origin from bacterial and archaeal plasmids [27,28], it has been hypothesized that some CRESS DNA viruses could infect prokaryotic hosts [9,29]. Endogenous sequences of CRESS DNA viruses have been found in the genomes of various eukaryotes, indicating a long history of interaction between these viruses and their hosts [30,31]. There are pathogens of poultry and pigs among CRESS DNA viruses that cause diseases with high mortality and significant economic losses [32,33,34,35]. These viruses have been repeatedly detected in feces, serum, cerebrospinal fluid, and respiratory secretions of patients with various diseases [24,36,37,38,39,40].

The prevalence of CRESS DNA viruses in animal populations and humans in Russia has not been studied. A single study examined such viruses in the plankton of Lake Baikal, identifying 12 partial CRESS-like genomes, many of which fall into distinct clusters specific to this lake [41]. In this study, the diversity of CRESS DNA viruses and alphasatellites was investigated in fecal samples from two widespread vole species—the northern red-backed vole (*Myodes rutilus*) and the tundra vole (*Microtus oeconomus*)—collected in the frequently visited green zone of the Novosibirsk metropolitan area.

## 2. Materials and Methods

### 2.1. Rodents and Samples

Samples of intestines with feces were collected from two animals that had been humanely euthanized—a northern red-backed vole (*M. rutilus*) and a tundra vole (*M. oeconomus*)—and stored at −80 °C. The animals (females) were humanely caught using traps in the Sovetsky District of Novosibirsk. The body mass at capture of the northern red-backed vole was 11.2 g, and the tundra vole was 31.8 g. All animal experiments were conducted in accordance with the Animal Welfare Act at the Omsk Research Institute of Natural Foci Infections and the guidelines for conducting experiments with laboratory animals (Supplement to the Order of the Russian Ministry of Health, no. 755, of 12 August 1977). This study was approved by the Bioethical Committee of the Omsk Research Institute of Natural Foci Infections (Protocol No. 4, 20 March 2013).

### 2.2. Viral Particle Purification and Nucleic Acid Extraction

Samples were homogenized in phosphate-saline buffer using a MagnaLyser (Roche, Basel, Switzerland) and further centrifuged at 15,000× *g* for 20 min. The supernatants were clarified by filtration, first through 0.45 µm and then through 0.22 µm filters to remove bacterial-sized particles. The samples were then subjected to ultracentrifugation to purify viral particles. For this purpose, an 80% sucrose solution was first placed in an ultracentrifuge tube, then layered with a 20% sucrose solution, and the filtered supernatants were applied on top. Centrifugation was performed for 2 h at 100,000× *g* at 4 °C. The layer at the interface between the 20% and 80% sucrose solutions was then collected and resuspended in PBS. The suspension was centrifuged for 1 h at 100,000× *g* and 4 °C. The pellet was resuspended in PBS and then treated with Turbo DNase (Ambion, Austin, TX, USA) according to the manufacturer’s instructions. Nucleic acids were then isolated using a standard phenol-chloroform extraction method [42]. The isolated DNA was amplified in a whole-genome amplification reaction using the REPLI-g Mini Kit (Qiagen, Hilden, Germany).

### 2.3. Viral Metagenome Library Construction for High-Throughput Sequencing

Fragment libraries of viral DNA and cDNA samples were generated and subjected to high-throughput parallel sequencing on the Illumina MiSeq platform [43]. Sequence contigs were then constructed in Unipro UGENE version 1.32.0 using the SPAdes algorithm [44,45]. For taxonomic assignment, the resulting contigs, along with singlets longer than 100 bp, were classified using optimal BLASTx values (E < 10^−5^) based on the GenBank nr database.

### 2.4. Genome Analysis

Reference viral sequences were obtained from GenBank. Nucleotide sequence alignment was performed using Clustal X version 2.0. [46]. Putative open reading frames (ORFs) in the genomes were predicted using the NCBI ORF finder program (https://www.ncbi.nlm.nih.gov/orffinder/, accessed on 1 February 2026). Circular structures of the genomes were predicted using Vector NTI 11.5 Advance (Invitrogen, Carlsbad, CA, USA). To identify hairpin structures, nucleotide sequences were analyzed using the Mfold server (https://www.unafold.org/, accessed on 5 February 2026) [47]. Amino acid sequence alignment was performed using Tcoffee (https://tcoffee.crg.eu/, accessed on 1 February 2026) [48]. Phylogenetic trees based on maximum likelihood were constructed using IQ-tree software version 2.0.5 [49]. Branch support was evaluated with 500 bootstrap iterations. The final trees were midpoint-rooted and graphically represented using FigTree version 1.4.1. The best-fit substitution model (VT + F + G4) was determined using IQ-Tee. GenBank accession numbers of the viral sequences used in the phylogenetic analysis are shown in the trees. The intron position was determined using the NetGene2 Server program (https://services.healthtech.dtu.dk/services/NetGene2-2.42/, accessed on 10 February 2026).

### 2.5. GenBank Sequence Numbers

The nucleotide sequences of the genomes obtained in this work were deposited in GenBank under accession numbers from MK738131 to MK738144.

## 3. Results

### 3.1. Genome Identification and Classification

**Figure 1:**
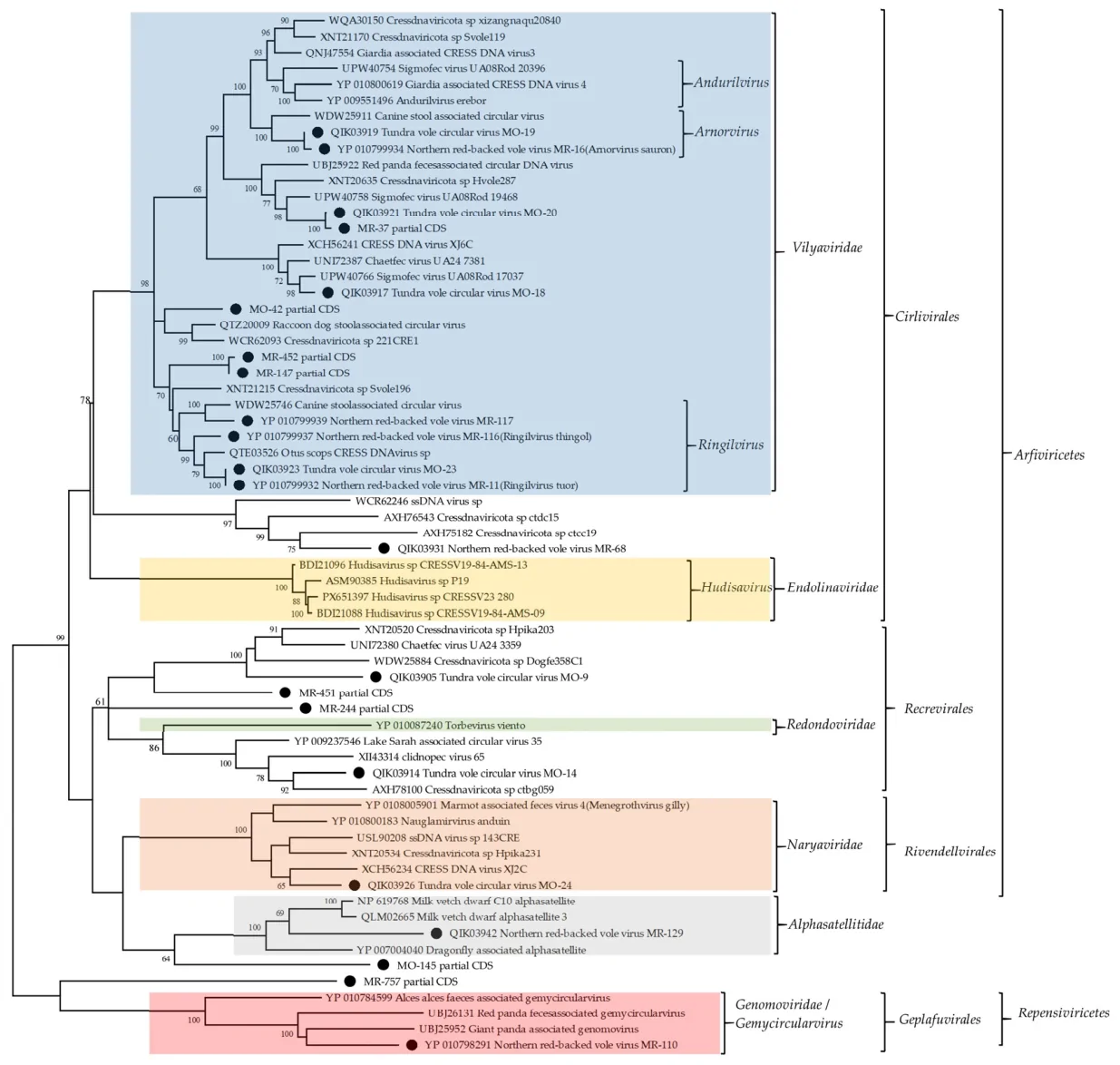
Maximum likelihood phylogenetic tree of the phylum Cressdnaviricota, based on the amino acid sequences of the Rep protein. Full-genome sequences are highlighted by black circles, and partial sequences by white squares. Different viral families are highlighted in different colors. Bootstrap values calculated from 500 replicates are given at the nodes. The sequence obtained from the raw human virome sequencing data (NCBI Sequence Read Archive, BioProject accession number PRJNA1521393, sample accession number SRR40427672) is marked with an asterisk.

Analysis of high-throughput sequencing data from *M. rutilus* and *M. oeconomus* intestines revealed 22 nucleotide sequences of varying lengths, classified as CRESS DNA viruses and alphasatellites based on the similarity of their encoded proteins to the Rep protein (Figure 1). The analysis used Rep sequences from GenBank with an identity of more than 60%. Thirteen nucleotide sequences were obtained from the *M. rutilus* virome (designated as MR) and nine sequences from the *M. oeconomus* virome (designated as MO). Analysis of the 22 sequences revealed that 14 of them represent circular complete genomes belonging to various families and unclassified CRESS DNA viruses and alphasatellites: seven from *M. rutilus* and seven from *M. oeconomus*. The remaining eight sequences were partial genomes.

**Figure 2:**
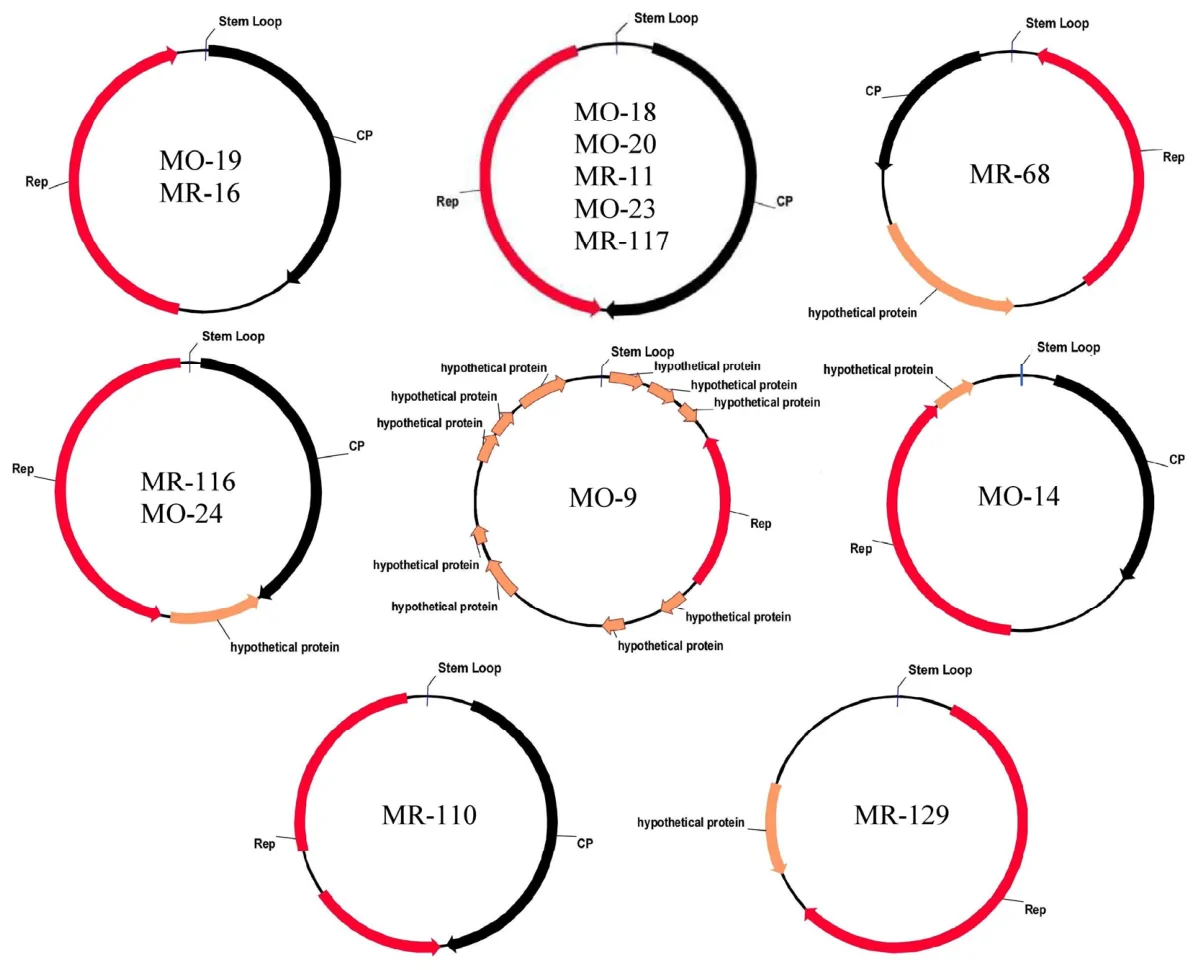
Comparison of the genome organization of the CRESS DNA virus. The *rep* genes are highlighted in red; *cp* genes are highlighted in black; and hypothetical ORFs are highlighted in orange. The isolates are described in Table 1.

The sizes of the complete genomes ranged from 1944 nucleotides (nt) to 4767 nt, and that of the alphasatellite was 1673 nt (Table 1). A nonanucleotide motif associated with *ori* (Table 1) and the *rep* genes was identified in all genomes, while the *cp* genes of CRESS DNA viruses were not found in two complete genomes MO-9 and MR-129 (Table 1). Pairwise amino-acid identity values varied significantly between the Rep sequences of CRESS DNA viruses and alphasatellites (Table 2). Hypothetical ORFs were detected in four genomes (Figure 2). A study of the genome organization of CRESS DNA viruses and alphasatellites allowed us to identify eight groups of genomes (Figure 2).

### 3.2. Taxonomic Assignment of the Genomes

The current taxonomy of CRESS DNA viruses and alphasatellites is based on the highly conserved Rep protein [12,50,51,52,53,54,55]. A phylogenetic analysis was performed on 22 Rep protein sequences of the studied CRESS DNA viruses and alphasatellites using related protein sequences from the GenBank database (Figure 1). As a result, 15 sequences were assigned to known families of the phylum *Cressdnaviricota*, whereas seven (MR-68, MO-9, MO-14, MR-451, MR-244, MO-145 and MR-757) were new (Figure 1).

Among 22 complete (*n* = 14) and incomplete (*n* = 8) genomes, 18 sequences were assigned to the *Arfiviricetes* class, whereas only one sequence belonged to the *Repensiviricetes* class. This taxonomic assignment was supported by analysis of conserved RCR and SF3 helicase motifs in the Rep protein sequence (Table 3). It has previously been shown that these motifs differ for CRESS DNA virus classes [12,13,14,19,20,21,22]. In addition, one sequence was a member of the *Alphasatellitidae* family and two partial sequences could not be assigned to the known classes or orders of CRESS DNA viruses (Figure 1).

### 3.3. Genomes Assigned to the Arfiviricetes Class

The eighteen *Arfiviricetes* class genomes were divided into three orders based on the Rep protein: *Cirlivirales* (*n* = 13)*, Recrevirales* (*n* = 4)*,* and *Rivendellvirales* (*n* = 1) (Figure 1).

Twelve of the 13 *Cirlivirales* genomes belonged to the *Vilyaviridae* family—eight complete genomes and four partial sequences. Of these, two complete genomes were assigned to the *Arnorvirus* genus, four to the *Ringilvirus* genus, and the remaining two complete genomes, MO-18 and MO-20, as well as four partial sequences, formed branches in taxonomically undescribed genera of the *Vilyaviridae* family (Figure 1). The other complete genome, MR68, was a member of an independent, taxonomically indeterminate cluster within the *Cirlivirales* order.

The eight complete genomes, MO-18, MO-19, MO-20, MO-23, MR-16, MR-11, MR-116, and MR-117, of the *Cirlivirales* order were assigned to the *Vilyaviridae* family with high reliability; their genome lengths varied from 1944 to 2295 nucleotides. The organization of these genomes differed: in isolates MO-19 and MR-16, the Rep and capsid protein genes were unidirectional, while these genes were located in opposite orientations in the remaining six isolates (Figure 2). Within the *Vilyaviridae* family, complete genome isolates MO-19 and MR-16 were assigned to the genus *Arnorvirus*, with MR-16 renamed *Arnorvirus Sauron* by the International Committee on Taxonomy of Viruses. Isolates MO-23, MR-11, MR-116, and MR-117 were assigned to the genus *Ringilvirus*, with MR-11 and MR-116 also renamed *Ringilvirus Tuor* and *Ringilvirus Thingol,* respectively, by the International Committee on Taxonomy of Viruses.

Notably, isolates MO-19 and MR-16 of the genus *Arnorvirus*, as well as MO-23 and MR-11 of the genus *Ringilvirus*, were detected in different rodent species, but have similar nucleotide sequences. Their genomes differ only by the presence of indels; isolate MR-16 of the species *Arnorvirus Sauron* differs from MO-19 by the presence of two deletions in the *rep* gene: the first is a single-letter deletion; the second is a two-letter deletion, 42 nucleotides apart, restoring the amino acid sequence of the Rep protein. Isolate MR-11 of the *Ringilvirus Tuor* species differs from MO-23 by the presence of a 101-nucleotide deletion in the intergenic sequence, near the nonanucleotide motif.

Isolate MR-68 of the *Cirlivirales* order does not belong to a known viral family and shows similarity to CRESS DNA viruses found in tissue of the red snapper (*Pristipomoides filamentosus*) (USA, ctcc19), Rainbow trout (*Oncorhynchus mykiss*) (USA), and Amur tiger (China).

In addition to the *Cirlivirales*, two complete genomes, MO-14 and MO-9, and two partial genomes were members of taxonomically undescribed genera/families of the *Recrevirales* order and one genome, MO-24, belonged to the *Rivendellvirales* order, *Naryaviridae* family (Figure 1). Both *Recrevirales* genomes occupied a distant branch on the tree constructed from the Rep protein sequences. Interestingly, the MO-9 genome is characterized by an abnormally low GC content—31% (Table 1). In addition, the genome organization of MO-14 and MO-9 substantially differed (Figure 2): MO-14 had the unidirectional Rep and capsid protein genes with an additional short hypothetical ORF, whereas MO-9 did not have the capsid protein ORF, but contained ten hypothetical ORFs (Figure 2), which did not demonstrate similarity with gene sequences from GenBank. Additional attempts to find the capsid protein gene, taking into account possible suppressed stop codons, were unsuccessful. Moreover, the MO-9 genome is unusually long, with 4767 nucleotides. All these results indicate that MO-14 and MO-9 belong to different taxonomic groups within the *Recrevirales* order.

### 3.4. Genomes Assigned to the Repensiviricetes Class

**Figure 3:**
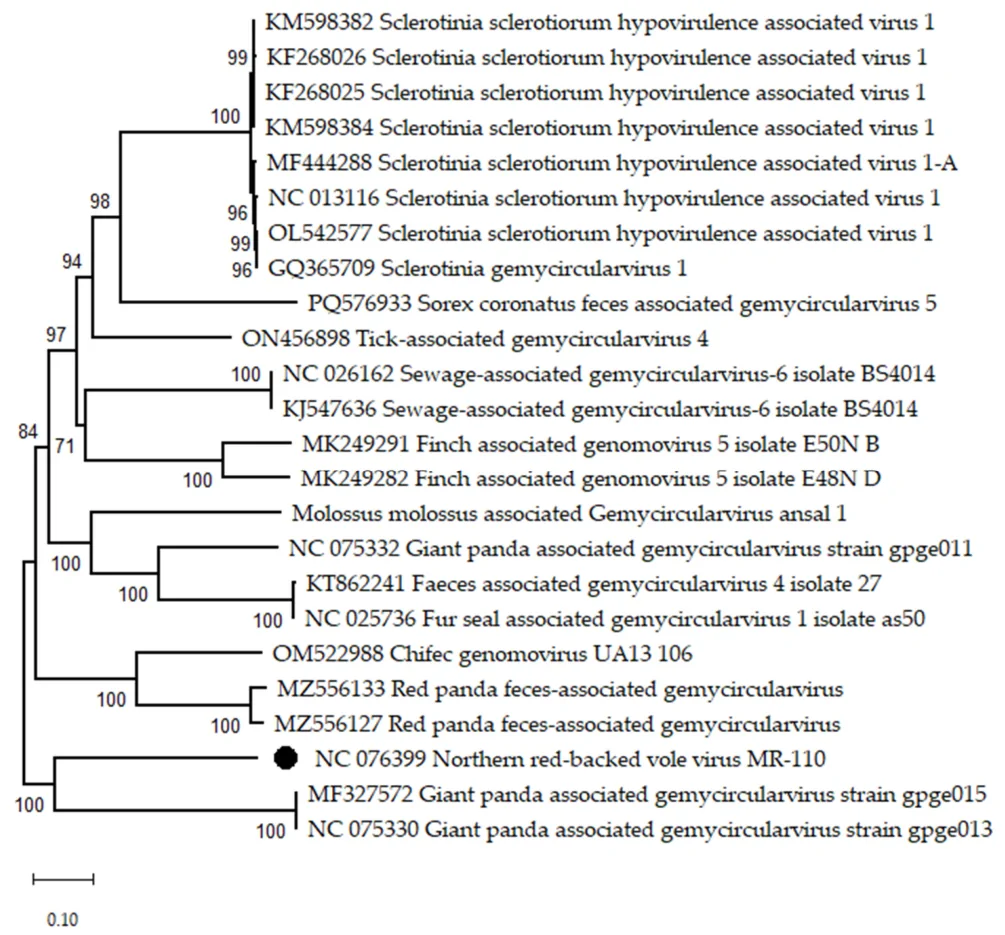
Maximum likelihood phylogenetic tree based on the complete genomes of viruses from the *Gemycircularvirus* genus. The sequence detected in this study is highlighted with a black circle. Bootstrap values calculated from 500 replicates are given at the nodes. The scale bar represents the number of substitutions per site.

Analysis of the constructed phylogram (Figure 1) allowed us to conclude that the MR-110 virus genome belongs to the gemycircularviruses (*Genomoviridae* family, *Repensiviricetes* class). The size of the MR-110 genome, 2.214 kb, corresponds to those of other gemycircularviruses, which range from 2.1 to 2.3 kb in length. The MR-110 genome, like other gemycircularviruses, contains a characteristic nonanucleotide motif TAATATTAT (Table 1). A phylogenetic analysis of sequences related to MR-110 was performed; for this purpose, complete genomes of viruses with more than 50% identity of the Rep protein were downloaded from GenBank (Figure 3). The MR-110 genome, in terms of nucleotide sequence, showed the greatest similarity to the genomes of giant panda gemycircularvirus strains gpge013 and gpge015 from China, while the level of identity with these genomic sequences is only approximately 46%. The organization and length of the MR-110 genome confirm its assignment to the genus *Gemycircularvirus* (Table 1), as does the analysis of the conserved RCR and SF3 helicase motifs in the Rep protein sequence (Table 3). In addition, the phylogenetic tree of the *Gemycircularvirus* complete genomes reliably confirmed the MR-110 taxonomy.

### 3.5. The Genome Assigned to the Alphasatellitidae Family

Based on the constructed phylogeny and genome organization (the absence of the *cp* gene), the genome of isolate MR-129 belongs to typical alphasatellite genomes (Figure 1 and Figure 2, Table 1) and contains a hairpin structure with a loop before the *rep* gene, including the nonanucleotide motif TAGTATTAC, which is characteristic of alphasatellites (Table 1). Analysis of the conserved domains of the amino acid sequences of the Rep protein (Table 3) also indicates that MR-129 is an alphasatellite. The length of the MR-129 viral genome sequence is 1673 nt, which is larger than the typical size of the genomes of other alphasatellites, usually 1300–1400 nt. A putative ORF of 66 aa in length was found in the MR-129 genome, but the significance of this finding remains unknown. Additional alphasatellite ORFs have been described previously, but their functions are unknown [56].

## 4. Discussion

The role of CRESS DNA viruses in various biocenoses is poorly understood, and their taxonomic classification is under development. These viruses represent an important area of study due to their wide ecological distribution, significant impact on agriculture and the economy, and their underestimated role in human health and global biogeochemical processes [9,10,11,12,33,34,35,36,37,38,39].

Twenty-two eukaryotic virus sequences determined during this study were classified as CRESS DNA viruses, 14 of which represented circular complete genome sequences of these viruses (Figure 1 and Figure 2). Most of the sequences were assigned to the *Vilyaviridae* family of the *Cirlivirales* order. Members of this family have a high GC content of approximately 59% and are specific to protozoans of the *Giardia* spp. The high GC content of their genomes makes them a convenient marker for analyzing intestinal or wastewater viromes to search for traces of *Giardia* [57,58].

Analysis of CRESS DNA sequences of viruses in the *Vilyaviridae* family revealed that genomes differed only in the presence of indels. The presence of deletion variants of CRESS DNA viruses in different rodent species in the absence of nucleotide substitutions may indicate the important role of this type of genetic variation in the evolution of these viruses.

Isolate MO-24 was assigned to the *Naryaviridae* family of the *Rivendellvirales* order (Figure 1). *Naryaviridae* is a recently established family of viruses that specifically infect *Entamoeba* parasites. The family was discovered through metagenomic analysis of human stool samples. *Naryaviridae* genomes have a relatively low average GC content of approximately 37%, which is among the lowest observed in eukaryotic CRESS DNA viruses. Evidence of recombination between different genetic modules (*rep* and *cp* genes) is a hallmark feature of *Naryaviridae* [58]. *Vilyaviridae* and *Naryaviridae* are examples of CRESS DNA viruses specializing in eukaryotic parasites. The detection of these CRESS DNA viruses possibly indicates the presence of *Entamoeba* and *Giardia* in the intestines of the collected rodent individuals.

Isolates MO-9 and MO-14 likely belong to the *Recrevirales* order, which includes the taxonomically characterized *Torbevirus viento* virus of the *Redondoviridae* family (Figure 1). Both MO-9 and MO-14, with corresponding related viruses, are located on two monophyletic branches, which presumably represent new unclassified genera or even families (Figure 1). All known viruses of the *Redondoviridae* family have been detected in specimens from the human respiratory tract and oropharyngeal sites, where *Redondoviridae* was the second most common virus family, and these viruses have been associated with periodontitis and critical illness [59,60]. MO-14-related viruses were isolated from Minnow tissue (ctbg059), from *Musculium novaezelandiae* (Lake Sarah-associated circular virus-35), and from *Ortmanniana pectorosa* (clidnopec virus-65); most of the hosts of these viruses are freshwater inhabitants. MO-9-related viruses were isolated from canines in China (Dogfe358C1), from *Chaetodipus* in the US (Chaetfec virus UA24_3359), and from *Ochotona curzoniae* in China (Hpika203). All hosts are mammals from various parts of the world.

Despite both MO-9 and MO-14 genomes belonging to the *Recrevirales* order, they substantially differ in size, GC-content, and genome organization between themselves. Remarkably, related genomes that form a common clade with MO-9 are similar in size and also encode a large number of hypothetical ORFs; however, their genomes contain the *cp* gene, unlike MO-9, in which it was not detected, either due to its high divergence or its absence. In the latter case, this suggests a new possible alphasatellite. Notably, the *cp* gene is highly variable; however, hypothetical ORFs in the MO-9 genome range from 114 to 309 nucleotides in length, while *cp* genes of related viruses range from 517 to 549 nucleotides in length and do not show significant similarity to any MO-9 ORFs. A search for intron splice sites in the MO-9 genome was unsuccessful.

CRESS DNA viruses possess unique evolutionary characteristics, including high rates of recombination and mutation, which provide them with exceptional evolutionary potential. They are capable of horizontal gene transfer between each other and various organisms, influencing the evolution of living beings and forming the “viral dark matter” of genomes [61]. In a recent study, Ben Chéhida et al. discovered a chimeric CRESS DNA virus that was a typical alphasatellite based on analysis of its Rep protein but possessed a *cp* gene [62]. Possibly, some CRESS DNA viruses can lose the *cp* gene and switch to satellite existence, and conversely, alphasatellites can acquire the *cp* gene, transforming into CRESS DNA viruses, which suggests reconsidering the current strict division of Rep-encoding sequences into viral and satellite clades. Currently, alphasatellites refer to members of the *Alphasatellitidae* family, which are genetically distinct from CRESS DNA viruses. Probably, a separate group of Rep-containing alphasatellites should be established, which are closely related to CRESS DNA viruses.

The complete MR-110 genome sequence was assigned to *Gemycircularviruses*, *Genomoviridae* family (Figure 1 and Figure 3). Gemycircularviruses represent a diverse and genetically variable group of pathogens infecting a wide range of eukaryotic organisms. The current study is ongoing to establish whether gemycircularviruses represent true human pathogens or primarily environmental/commensal viruses [63,64,65,66,67,68,69]. Different gemycircularvirus strains show varying amino acid sequence identities when compared phylogenetically, often sharing only 30–50% identity with known strains, indicating that many novel species remain to be discovered. Based on the genome sequence alone, it is impossible to identify possible hosts of MR-110.

One explicit alphasatellite, the MR-129 genome, from the *Alphasatellitidae* family was identified in this study (Figure 1). This is a family of small, circular, ssDNA satellites frequently associated with geminiviruses and nanoviruses [15,70,71]. Alphasatellites can be divided into five clades based on the hairpin structures [72]. Some studies revealed complex roles for alphasatellites in the CRESS DNA viruses’ pathogenesis [73,74]. Alphasatellites demonstrate substantial genetic diversity and broad host plant ranges; they have been detected worldwide in association with various crop diseases and display distinct effects on their severity [75,76,77]. The studied MR-129 isolated from the intestine of a vole is possibly a satellite of an unknown CRESS DNA plant virus.

Taking into consideration that small rodents could be a reservoir of various zoonotic infections, all complete and partial CRESS DNA genomes obtained in this study were compared with genomes extracted from the viromes of volunteers living near the habitat of the collected rodents. No similar sequences were found. A single identified CRESS DNA virus sequence—*Hudisavirus* CRESSV23_280 (the *Endolinaviridae* family, *Cirlivirales* order), GeneBank #PX651397 was detected in a fecal sample of a healthy donor. This was a non-randomized pilot study evaluating the efficiency of fecal microbiota transplantation [78]. The pilot study was conducted in accordance with the Helsinki Declaration and approved by the Local Ethics Committee of the Autonomous Non-Commercial Organization “Center of New Medical Technologies in Akademgorodok” (protocol #1; 18 January 2018). This genome is close to those isolated from humans from the USA (P19) and the Netherlands (CRESSV19-84-AMS-09), indicating the widespread distribution of these viruses (Figure 1). Similar sequences from natural inhabitants have not yet been found. The *Hudisavirus* genus contains relatively recently discovered viruses and although these viruses have been detected in human fecal samples, their pathogenicity has not been studied, and they are currently classified as commensal viruses [79,80,81,82].

Detection of new CRESS DNA virus genomes indicates the importance of further study of virus evolution, the factors that influence their spread, and potential impact on ecosystems. Due to anthropogenic pressure, animals can alter their behavior and habitat use, which may lead to the spread of these viruses. This underscores the importance of studying the virome of synanthropic animals. Studying the viromes of wild rodents living near humans is useful for monitoring the circulation of known viruses and identifying new ones. Comparing this data with the human intestinal viromes can reveal the impact of anthropogenic factors on the ecology.

The limitation of this study is that only one sample from a species was used for the study. It does not allow conclusions about the prevalence and ecological significance of the viruses in the particular region. Nevertheless, the discovery of new genomes indicates that the list of CRESS DNA viruses is incomplete.

In conclusion, the study revealed the biodiversity of eukaryotic viruses in voles inhabiting the park zone of Novosibirsk. Complete CRESS DNA genome sequences analyzed in this study revealed both similar and unique sequences that differ from those presented in databases. This requires improved CRESS DNA viral taxonomy, especially for alphasatellites.

## Figures and Tables

**Table 1:** Complete genome sequences of CRESS DNA viruses and alphasatellites identified in this study.

№	Isolate	Genome Size (nt)	GC Content	Nonanucleotide Motif	Genbank Number	Family
1	MO-19	2210	56.56%	TAATACTAT	MK738134	*Vilyaviridae*
2	MR-16	2207	56.59%	TAATACTAT	MK738139	*Vilyaviridae*
3	MO-18	2295	58.17%	TATCATTTT	MK738133	*Vilyaviridae*
4	MO-20	2178	60.88%	CTACTTTCC	MK738135	*Vilyaviridae*
5	MR-11	1944	53.09%	TTATCGTGT	MK738138	*Vilyaviridae*
6	MO-23	2046	52.93%	TTATCGTGT	MK738136	*Vilyaviridae*
7	MR-117	2075	57.4%	TTTGATTAG	MK738143	*Vilyaviridae*
8	MR-116	2122	56.31%	GCCCCTACA	MK738142	*Vilyaviridae*
9	MR-68	4725	43.56%	TAATATTAC	MK738140	Unclassified
10	MO-9	4767	31.11%	AATCGAATC	MK738131	Unclassified
11	MO-14	2676	40.51%	TAATACTAT	MK738132	Unclassified
12	MO-24	1961	35.08%	CAAAATCAT	MK738137	*Naryaviridae*
13	MR-110	2214	51.31%	TAATATTAT	MK738141	*Genomoviridae*
14	MR-129	1673	46.68%	TAGTATTAC	MK738144	*Alphasatellitidae*

The order of the isolates in the table corresponds to that in Figure 1.

**Table 2:** The amino-acid identity between the Rep sequences of CRESS DNA viruses and alphasatellites identified in this study.

	MO-19, QIK03919	MR-16, YP010799934	MO-18, QIK03917	MO-20, QIK03921	MR-11, YP010799932	MO-23, QIK03923	MR-117, YP010799939	MR-116, YP010799937	MR-68, QIK03931	MO-9, QIK03905	MO-14, QIK03914	MO-24, QIK03926	MR-110, YP010798291	MR-129, QIK03942
MO-19, QIK03919	ID	0.953	0.426	0.410	0.436	0.436	0.396	0.444	0.141	0.159	0.221	0.174	0.118	0.150
MR-16, YP010799934	0.953	ID	0.405	0.387	0.409	0.409	0.369	0.419	0.132	0.148	0.208	0.163	0.110	0.144
MO-18, QIK03917	0.426	0.405	ID	0.345	0.371	0.371	0.335	0.365	0.133	0.162	0.202	0.176	0.121	0.141
MO-20, QIK03921	0.410	0.387	0.345	ID	0.356	0.356	0.322	0.351	0.138	0.126	0.203	0.143	0.106	0.125
MR-11, YP010799932	0.436	0.409	0.371	0.356	ID	1.000	0.621	0.693	0.127	0.164	0.218	0.173	0.127	0.148
MO-23, QIK03923	0.436	0.409	0.371	0.356	1.000	ID	0.621	0.693	0.127	0.164	0.218	0.173	0.127	0.148
MR-117, YP010799939	0.396	0.369	0.335	0.322	0.621	0.621	ID	0.596	0.126	0.167	0.208	0.182	0.118	0.145
MR-116, YP010799937	0.444	0.419	0.365	0.351	0.693	0.693	0.596	ID	0.135	0.168	0.201	0.157	0.118	0.139
MR-68, QIK03931	0.141	0.132	0.133	0.138	0.127	0.127	0.126	0.135	ID	0.078	0.101	0.074	0.078	0.076
MO-9, QIK03905	0.159	0.148	0.162	0.126	0.164	0.164	0.167	0.168	0.078	ID	0.157	0.156	0.076	0.107
MO-14, QIK03914	0.221	0.208	0.202	0.203	0.218	0.218	0.208	0.201	0.101	0.157	ID	0.161	0.084	0.127
MO-24, QIK03926	0.174	0.163	0.176	0.143	0.173	0.173	0.182	0.157	0.074	0.156	0.161	ID	0.093	0.117
MR-110, YP010798291	0.118	0.110	0.121	0.106	0.127	0.127	0.118	0.118	0.078	0.076	0.084	0.093	ID	0.068
MR-129, QIK03942	0.150	0.144	0.141	0.125	0.148	0.148	0.145	0.139	0.076	0.107	0.127	0.117	0.068	ID

**Table 3:** Conserved RCR and SF3 helicase motifs found in the Rep protein recovered in this study.

№	Isolate	RCR Motif			GRS	SF3 Helicase Walker-A	SF3 Helicase Walker-B	SF3 Helicase Motif C	Order
		I	II	III					
1	MO-19	CGTLNS	QHFQFY	YCTK	-	GESGSGKS	ILDDF	ITSN	*Cirlivirales*
2	MR-16	CGTLNS	QHFQFY	YCTK	-	GESGSGKS	RLGDF	ITSN	*Cirlivirales*
3	MO-18	AFTLFC	LHLQGF	YCTK	-	GPSGSGKS	VLDDF	ITSN	*Cirlivirales*
4	MO-20	CGTVND	RHLQFF	YCMK	-	GPSGTGKS	VLDDF	LTSN	*Cirlivirales*
5	MR-11	CGTLQL	IHFQFY	YCTK	-	GDTGTGKS	IFDDF	FTSN	*Cirlivirales*
6	MO-23	CGTLQL	IHFQFY	YCTK	-	GDTGTGKS	IFDDF	FTSN	*Cirlivirales*
7	MR-117	CGTLQL	AHLQFY	YCSK	-	GATGTGKS	IFDDF	FTSN	*Cirlivirales*
8	MR-116	CGTLQL	LHFQFY	YCTK	-	GDTGAGKS	IFDDF	FTSN	*Cirlivirales*
9	MR-68	IFTWNN	PHFQGY	YCTK	-	GPTAVGKT	LMDEF	FATN	*Cirlivirales*
10	MO-9	MGTIWS	EHWHCY	YCKS	-	GPAGCGKT	LMDDV	VTSN	
11	MO-14	VFTLNN	SHLQGA	YCTK	-	GETGTGKT	IFDDF	ITCP	*Recrevirales*
12	MO-24	QLTLNE	EHIHIY	YIRK	-	GKSGTGKS	IYDDF	ITSI	*Rivendellvirales*
13	MR-110	LLTYAQ	IHLHAF	YAVK	NVFDVQGHHPNIEKSR	GPTRLGKT	IFDDI	WLAN	*Geplafuvirales*
14	MR-129	CFTLNN	PHYQGF	YAKK	-	PLGGEGKT	LVIDI	TSTK	-

The order of the isolates in the table corresponds to that in Figure 1.

## Data Availability

The original contributions presented in this study are included in the article. Further inquiries can be directed to the corresponding authors.
